# Physicochemical Characteristics of Bambara Groundnut Speciality Malts and Extract

**DOI:** 10.3390/molecules27144332

**Published:** 2022-07-06

**Authors:** Adeola Helen Adetokunboh, Anthony O. Obilana, Victoria A. Jideani

**Affiliations:** Department of Food Science and Technology, Cape Peninsula University of Technology, Bellville 7535, South Africa; helinaa2002@gmail.com (A.H.A.); obilanaa@cput.ac.za (A.O.O.)

**Keywords:** Bambara groundnut, α-amylase, β-amylase, total polyphenols, antioxidant, steeping, sprouting, metabolites

## Abstract

Speciality malts and their extracts have physicochemical characteristics such as colour, flavour, and aroma sorted for in food production. Speciality malts used in food production are mostly produced from cereal grains. Hence, this study aimed to produce speciality malts from Bambara groundnut (BGN) seeds and analyse their physicochemical characteristics and metabolites. The base, toasted, caramel, and roasted malt were produced by drying at different temperatures and times. Syrups were produced isothermally from the speciality malts. The speciality malts and syrups were assessed for colour, pH, protein, α and β-amylases, total polyphenols, antioxidants, and metabolite profiling. The BGN speciality malts were assayed for fatty acid methyl esters (FAME), hydrocarbons, sugar alcohols, sugars, acids, amino acids, and volatile components using capillary gas chromatography-mass spectrometry (GC-MS) and gas chromatography with flame ionisation detection (GC-FID). The colours of the speciality malts and syrups were significantly (*p* = 0.000) different. The protein content of the BGN speciality malts was significantly different (*p* = 0.000), while the protein content of the syrups was not significantly different. The amylase activities of the BGN speciality malt decreased with the change in kilning temperatures and time. The α- and β-amylase activities for the specialty malts were 1.01, 0.21, 0.29, 0.15 CU/g and 0.11, 0.10, 0.10, 0.06 BU/g. The total polyphenols and antioxidant activities differed for all BGN speciality malts. There were twenty-nine volatiles detected in the BGN speciality malts. Fifteen amino acids consisted of seven essential amino acids, and eight non-essential amino acids were detected in the speciality malts. Fatty acid methyl esters (FAME) identified were palmitoleic, oleic, linolelaidic, linoleic, and arachidic acid. The sugars, organic acids, and sugar alcohols consisted of lactic acid, fructose, sucrose, and myo-inositol. The BGN speciality malts exhibited good physicochemical characteristics and metabolites that can make them useful as household and industrial ingredients for food production, which could be beneficial to consumers.

## 1. Introduction

Household processing such as dehulling, boiling/cooking, pressure cooking, milling, roasting, fermentation, soaking, and malting are applied to improve the physicochemical properties of cereals and legumes [1,2,3,4]. Malting is an inexpensive household food processing method that has recently gained attention from researchers to study legumes [4,5,6,7,8]. Malting consists of three simple steps: steeping, sprouting, and drying under controlled conditions [9,10].

Malting of legumes has been reported to encourage an increase in free amino acids and vitamins by the modification of the functional properties of the seed’s physical and chemical components [11,12,13]. In addition, malting promotes hydrolytic enzymes, which are not present in ungerminated grains [14,15]. Due to the activation of the hydrolytic enzyme, the malting process (soaking, sprouting, and kilning) gives malted grains their characteristic colour, taste, flavour, and nutritional components [16,17,18].

The final step of malting, kilning, is a biochemical process applied to cereals and legumes to enhance their physicochemical properties. The kilning temperature and time are increased to obtain desired malt properties such as enzymes, moisture removal for stabilisation, raw flavours removal, malty flavours, and colour development [19,20,21,22,23]. During the kilning process, the reaction of sugars and amino acids promotes melanoidins and reductones through the Maillard reaction [19,24,25,26]. The melanoidins formed are responsible for the antioxidant potential of the speciality malt types [21,24,26,27]. For example, the dark speciality malts’ Maillard reaction products (melanoidins) are significant antioxidants that increase with increasing malt colour due to changes in kilning temperature and time [23,25,28].

Producing speciality malts comes with different drying temperatures [24,29,30]. Base malts are produced at a low temperature between 50 and 80 °C for their high degree of diastatic power. The base malt mostly precedes other speciality malts such as roasted, toasted, and caramel malts used in food processing for various benefits [24,31,32]. Caramel, roasted, and toasted malts are termed speciality malts because they are produced primarily because of their characteristic high antioxidants, colour, and flavour [28,30,32,33]. Generally, speciality malts and their extracts (syrup) add sensory benefits to the final product by enhancing their colour, flavour, and taste [34,35].

Barley is the most used ingredient in malt production, and it is majorly used in the brewing and food industries [36]. Although barley malt is commonly used, other cereals and legumes are also malted to access their nutritional value [37]. Mung beans, soybeans, cowpea, black beans, lentils, chickpea, and Bambara groundnut are some of the legumes that have been sprouted and studied for their nutritional, physicochemical, and functional characteristics [38].

Bambara groundnut (BGN) is not a commonly known legume crop in many parts of the world. However, it is categorised as the third most crucial legume in Africa, after peanuts and cowpeas [39,40]. Sustainable food experts have gained interest in BGN because it is an underutilised and nutritious crop [41]. BGN is an indigenous plant cultivated in Africa on a small scale by subsistence farmers [42,43]. It is a good quality protein food containing substantially high proteins, carbohydrates, fats, and minerals [13,40].

Following the malting process, BGN seeds’ physicochemical, functional, thermal, health-promoting, and nutritional properties greatly improved while reducing their anti-nutritional factors [13,15]. Abba et al. [44] noted that malting BGN improved its protein content. Value-added snacks, weaning, ready-to-eat, and composite products have been made from malted BGN seeds and have shown improvement over the unmalted seeds [45,46]. The nutritional and functional characteristics of malted BGN in the production of *Okpa*, composite biscuits, flours, and infant formula, have been investigated [46,47], where the malted BGN samples were acceptable to consumers. Bambara groundnut was subjected to steeping (36 and 48 h) and sprouting (0 to 144 h) at different times to study its physicochemical characteristics [48]. The study reported that the BGN malt exhibited good physicochemical characteristics peculiar to malt products and can be used as a functional food ingredient in food and beverage formulations [48]. In this study, the amylase-rich BGN malt produced by steeping for 36 h and sprouting for 96 h was used to produce speciality malts. However, no documented study has reported the physicochemical characteristics of BGN speciality malts and syrup products. Thus, this work investigated the production of BGN speciality malts and syrups, their physicochemical characteristics, and metabolites.

## 2. Results and Discussion

### 2.1. Colour Characteristics of Bambara Groundnut Speciality Malts and Syrups

**Figure 1:**
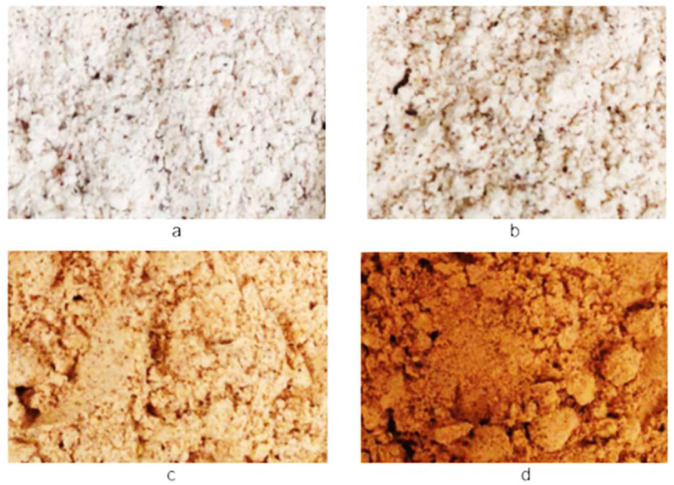
Bambara groundnut speciality malts: (**a**) base malt, (**b**) caramel malt, (**c**) roasted malt, and (**d**) toasted malt.

The CIE L*a*b* colour space coordinates, chroma, and hue of the BGN speciality malts consisting of base (BM), caramel (CM), roasted (RM), and toasted (TM) malts are shown in Table 1. The lightness (L*) and the hue angle (h°) decreased from 74.12 to 45.98 and 71.54 to 53.90 for the BGN speciality malt types. The redness (a*), yellowness (b*), and chroma increased for the BGN speciality malts, 3.96 to 16.44, 11.85 to 22.68, and 12.50 to 28.03, respectively. There was a significant difference across the lightness, redness, yellowness, chroma, and hue for all the speciality malts. As seen by the physical eye, the colour of the speciality malt was as shown in Figure 1.

The colour change has been attributed to the non-oxidative Maillard reaction due to heat [32]. The reaction between reducing sugars and amino acid contents of malted grains consists of the Maillard reaction’s complex reactions [49]. The reactions are important mechanisms of non-enzymatic browning during heat processing of malt [50]. The factors affecting the degree and magnitude of the Maillard reaction are temperature, time, water activity, and concentration [32]. These factors affect the end product and give the products their characteristic colour, flavour, and anti-oxidative activity, which are essential in industrial food products [51].

The colour of the malt is greatly affected by temperature and time [52]. Yahya et al. [53] reported a similar result in the production of barley and malt roasting operations where the product became darker as temperature increased above 150 °C, showing lightness (L*) reducing from 75 to 40. Furthermore, the evaluation of the colour coordinates in the study of barley speciality malt features showed a colour difference, whereby there was a decrease in lightness (L*) at high temperatures, while the redness (a*) and yellowness (b*) increased at higher temperatures [33]. Studies on the development of Maillard reaction during roasted (caramel) malt production demonstrated that the colour formation depends mainly on the time and temperature of kilning [24,28,54].

However, the colour changes were attributed to the measure of shorter chain melanoidins or caramelisation by conversion to darker-coloured malt with increased temperatures [22]. The base, caramel, roasted, and toasted malt syrup’s lightness values are indicated in Table 2. The chroma and hue angle (h°) for the base, caramel, roasted, and toasted malt syrups ranged from 8.68 to 21.75 and 59.20 to 73.53°, respectively. The hue angle (h^o^) of the BGN syrups represents the red to yellow colour range (0°–90°). There is, however, a significant (*p* = 0.000) difference in the speciality malt syrup’s lightness, redness, yellowness, and chroma, except for hue. The higher colour values signify the BGN speciality malt syrup colour’s intensity. The reduction in lightness of the syrups from base malt syrup to toasted malt syrup was attributed to the Maillard reaction developing Maillard reaction products during heating and caramelisation [55,56]. The parameter redness (a*) and yellowness (b*) were positive values indicating reddish and yellowish syrup colours. The redness was highest for the roasted malt syrup, and the yellowness was lowest for toasted malt syrup, attributed to the differences in the kilning temperatures and time [57]. The BGN speciality malts syrups’ chroma was lowest for the toasted BGN speciality malt syrup. The chroma values refer to the colour saturation, where low chroma values are weak, and high chroma values are highly saturated or strong [58,59]. The chroma values for the study on rice syrup were low, with a dark brown colour range compared to this study [25,60].

**Figure 2:**
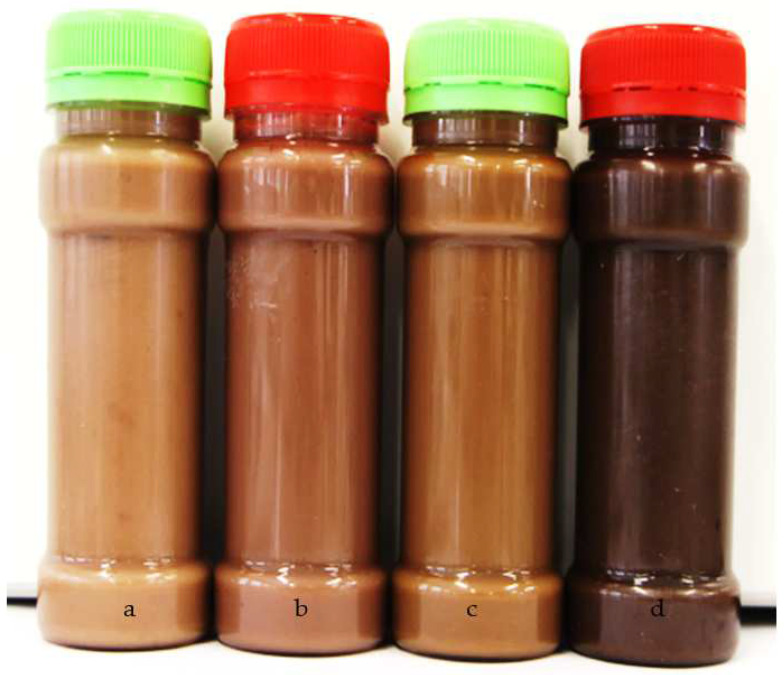
Bambara groundnut speciality malt syrups: (**a**) base malt syrup, (**b**) caramel malt syrup, (**c**) roasted malt syrup, (**d**) toasted malt syrup.

The speciality malts’ hue angle was higher than that of the syrups due to heat application during mashing. Hue angle (h°) is the attribute of a colour distinguished by the red, yellow, blue, green, or purple object [58]. Hue angle (h°) can accurately determine how humans perceive colour, as shown in Figure 2 [60]. The BGN speciality malt syrup hue angle range of 59.20 to 73.53° indicated reddish-yellow. The hue angle (h°) of the speciality malt syrup is consistent with other studies that reported a decrease in h° value during heat application to syrup [57,58]. In addition, the colour of the BGN speciality malt syrup indicated that more colour developed during the wort boiling based on temperature and time [61].

Malt extract boiling generally increases wort colour due to the formation of melanoidins, the caramelisation of sugars, and polyphenols’ oxidation [62]. The application of heat reduced the lightness (L*), redness (a*), yellowness (b*), and the hue of the syrup for all BGN speciality malt syrups, while hue angle (h°) increased. Moreover, the toasted malt syrup exhibited the darkest colour for the colour parameters (CIE L*a*b*, chroma). The decrease in lightness, redness, and yellowness of speciality malt syrup has been attributed to the formation of colour compounds (melanoidins) due to the Maillard reaction during kilning and further heating when producing syrups, thus providing desirable colours to food produced with them [60].

The speciality malt, extracts, and syrups are good sources of natural colour enhancement in food industries for beverages, baked items, and culinary recipes [63,64]. The colour enhancement can be attained using a base malt ratio with specialised malt flours, malt extracts, or syrup [63,65]. The speciality malts and syrups in this study exhibited colours desirable in the food industries, which could be used to impact the colours of baked goods and breakfast meals similar to the popular barley malt [22]. Furthermore, being significantly different could mean that the speciality malts and syrups could impact different shades of colours as ingredients in product formulation.

### 2.2. The pH Characteristics of the Bambara Groundnut Speciality Malts and Syrups

**Figure 3:**
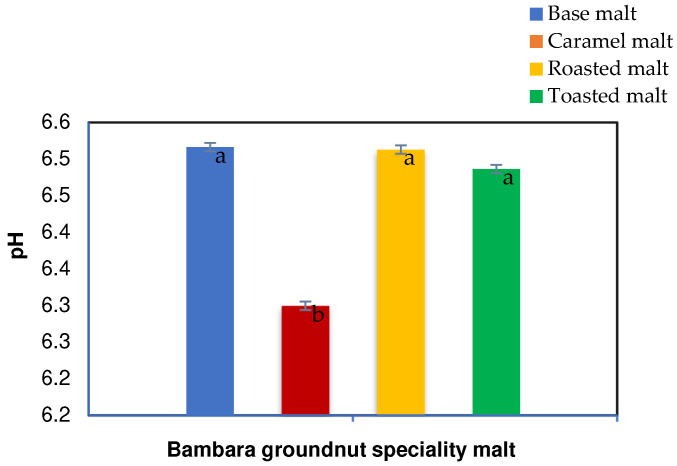
pH characteristics of Bambara groundnut speciality malt. Values are mean ± standard deviation of triplicate values; mean values on the bars with different letters are significantly different (*p* < 0.05).

The pH for the BGN speciality base (BM), caramel (CM), roasted (RM), and toasted (TM) malts ranged from 6.30 to 6.52. The base, roasted, and toasted malts were not significantly different, as shown in Figure 3. However, the base, roasted, and toasted malts exhibited higher pH than the caramel malt. The caramel malt had the lowest pH value, while the higher pH in the three speciality malts has been attributed to variations in temperature and time [33]. However, the high pH of the base malt is a characteristic of base malt [66]. During their study, Vandecan et al. [33] showed that the time and temperature of roasting caramel malt resulted in a pH increase. Their results showed that malt pH decreased with increasing kilning temperature due to the acidic Maillard reaction products, reductones and melanoidins. However, there was a pH increase after the initial decrease with increased roasting temperature to 180 °C, similar to this study. The pH increase was ascribed to the decline in the concentration of acidic components due to evaporation, further conversion, and polymerisation reactions [24,33]. In their study, Geurts [67] noted that the malt pH depends on the production method used to create speciality malt. The effects of pH on speciality malts have been studied, where it was discovered that dark malts tend to exhibit higher pH than pale (base malt) and light caramel malt [67,68]. The high pH is due to the dark roasted and toasted malt products being roasted at high temperatures that are enough to use Maillard reaction, caramelisation, and pyrolysis, which can affect the pH of the speciality malt [67,69].

The characteristic pH values of the BGN speciality malt syrups for the base, caramel, roasted, and toasted malt syrups were 5.52, 5.13, 5.46, and 5.71, respectively, in Table 3. The pH of the toasted malt syrup was much higher than the base, caramel, and roasted malt syrups, with a significant (*p* = 0.000) difference. pH is crucial in wort production; it regulates the activity of the enzymes (external and internal) in the mash [70]. The mashing and wort boiling period is the application of heat treatment that can separate the calcium ion (Ca^2+^) bound with phosphates (K_2_PO_4_) and polypeptides to form insoluble compounds by the release of hydrogen ion (H^+^) and decrease the wort pH [70,71,72,73]. Due to boiling, the wort becomes acidic with a range of 0.1–0.3 pH units for a typical boiling process due to the melanoidins formation [61,62]. Moreover, the pH of the BGN caramel, roasted, and toasted speciality malt syrups was lower and was attributed to the formation of acids from sugars compared to the base malt syrup [62,67,72].

Due to heat application during boiling, the pH is relatively low after malt syrup production [73]. The pH values of the wort produced from chickpea, yellow pea, common vetch, and green lentil were 5.44, 5.7, 5.53, and 5.51, respectively [74]. These are in the same range as this study’s BGN speciality malt syrup. The pH of the BGN speciality malt syrups is in the same range as that of the barley malt syrups under study in the specific European brewery convention range [74,75,76,77]. The BGN speciality malt syrups exhibiting a similar pH range might make them useful in brewing industries as a substitute for malted barley. Thus, producing BGN malt syrups isothermally, as described in this study, produced products that could be used in product formulation, promoting BGN as a functional ingredient.

### 2.3. The Protein Content of Bambara Groundnut Speciality Malts and Syrups

**Figure 4:**
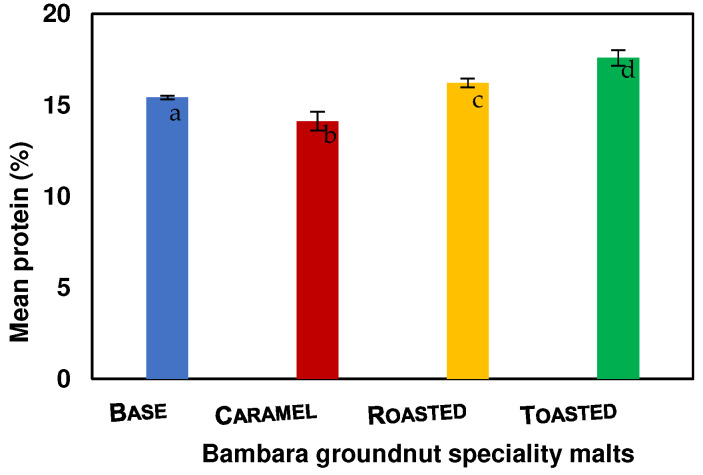
Protein content of Bambara groundnut speciality malts. Values are the mean of triplicates ± standard deviation; mean values on the bars with different letters are significantly different (*p* < 0.05).

The protein content values of the base, caramel, roasted, and toasted BGN speciality malts are 15.41, 14.12, 16.22, and 17.58, respectively, as shown in Figure 4. The protein contents for the BGN speciality malts are significantly (*p* = 0.000) different, with caramel malt having the lowest protein content. The difference in the protein content could be due to different kilning temperatures and times. It was noted that high kilning temperatures of malted HomChaiya rice influenced the protease enzymes similar to this study, which invariably increased the soluble protein and amino acids [51]. Therefore, the increase is attributed to the soluble protein as kiln temperature increased due to an acceleration of proteolytic activities.

In contrast to the results of this study, Diedericks et al. [40] reported a reduction in the protein of BGN seeds subjected to roasting from 70 to 179 °C for soaked and unsoaked BGN seeds. They attributed the reduction to the exposure to high temperature due to denaturation of proteins depending on their thermal stability. However, [78] recorded no difference in protein content at different temperatures up to 100 °C in Greek barley.

**Figure 5:**
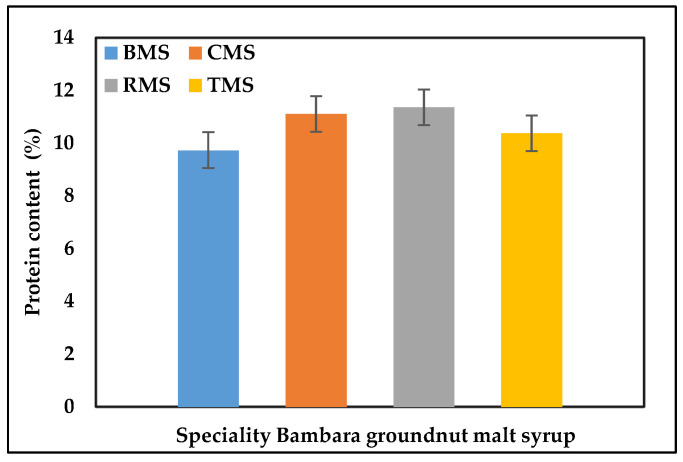
The protein content of the BGN speciality malt syrup, BMS—base malt syrup, CMS—caramel malt syrup, RMS—roasted malt syrup, TMS—toasted malt syrup.

The Bambara groundnut speciality malt syrups protein content was lower after mashing and boiling, ranging from 9.73, 10.37, 11.10 to 11.35 for the base, caramel, roasted, and toasted malt speciality syrups, respectively, as shown in Figure 5. Based on the Kruskal–Wallis test, protein distribution was the same across the syrups, showing no significant difference. However, [74,79,80] produced gluten-free worts and malt extracts from legumes, resulting in high protein content with reduced anti-nutritional constituents and increased antioxidants. Wort boiling is a thermal process whereby various chemical, physicochemical, physical, and biochemical reactions occur. The boiling of the wort is important for sterilising the wort, stopping enzymatic reactions, water evaporation from the wort, unwanted aroma compounds removal, and hot break or hot trub, which is the precipitation of the wort protein contents’ insoluble coagulum [62,81].

The protein content of malt is dependent on the enzyme-to-substrate ratio, that is, the ratios of α- and β-amylases/starch and endo-peptidases/proteins [62]. The protein contents of the malt extract decrease after boiling, which matches the results reported by [82] and [83]. The reduction in protein can be attributed to protein degradation during mashing and wort boiling [84,85]. In contrast to the reduction in protein observed in this study and literature, [83] established that the barley wort protein content increased, and this was ascribed to the elevated stability of the soluble proteins. However, the BGN speciality malt syrup exhibited a good proportion of protein content that could benefit consumers.

### 2.4. Amylase Activities of Bambara Groundnut Speciality Malts and Syrups

The α and β-amylase activities for the base, caramel, roasted, and toasted BGN speciality malts were 1.01, 0.21, 0.29, 0.15 CU/g and 0.11, 0.10, 0.10, 0.06 BU/g, respectively, as shown in Table 4. The amylase activities of the BGN speciality malts differed significantly (*p* = 0.000) across the malt types. Studies have shown that amylase activities change with changes in kilning temperature and time, which is similar to this study’s results [36,51,86,87]. Kilning temperature and time of the germinated sorghum grains reduced the α- and β-amylase activities [88]. Uriyo [89] observed that kilning black-eyed peas at higher temperatures reduced the α-amylase activities, and β-amylase activity could not be detected in the germinated cowpea. The α and β-amylase of cowpea, buckwheat, sorghum, teff, and barley malt were found to decrease linearly with an increase in drying temperature [87,90,91,92]. As with other studies on sorghum, buckwheat, teff, barley, and cowpea, β-amylase was low or absent after kilning, which correlates with this study on BGN speciality malt [89,93]. In addition, α- and β-amylase decreased with a change in kilning temperature and time, where the β-amylase showed the lowest value. The resultant reduction in α and β-amylase regarding kilning temperature and time was because diastatic enzymes can only survive in mild kilning due to the formation of heat-stable complexes in the starch granules [94]. The decrease in the enzymatic activity could thus be due to the heat denaturation of grains, known as the enzyme-inactivating phase [86,90,95]. The barley α-amylase is more thermostable than β-amylase; the α and β-amylase of the BGN speciality malts have shown similar thermostability [87,91]. There was an increased inactivation by kilning due to denaturation by heat application [87,95,96]. Despite the heat application during kilning, mashing, and boiling of malt wort to produce syrup, some amylase survives [94,97,98,99].

The base, caramel, roasted, and toasted BGN speciality malt syrups (BMS, CMS, RMS, and TMS) α-amylase values were 0.39, 0.31, 0.30, 0.31 CU/g, and β-amylase values were 0.14, 0.13, 0.15, 0.21 BU/g, respectively, as shown in Table 5. There is a significant (*p* = 0.000) difference across the amylase activities of the speciality malt syrups. The increase in the α and β-amylase activities observed in the BGN speciality malt syrups after wort boiling is due to the activities of the enzymes [61,100]. The production of malt-based syrups involves producing the malt, the mashing process to produce wort from the malt, and the concentration of the wort to malt syrup by boiling [74,101]. Characteristics of malt syrup are brown, sweet, gluey liquids with diastatic enzymes (base malt) or without diastatic enzymes (speciality malt) [102]. Speciality malts are very important for enhancing and improving malt wort (syrup) by improving its colour and flavour [102].

The α-amylase activity of the base malt syrup was the highest, while the roasted malt syrup activity was shown to be the lowest. The β-amylase is thermally unstable; it is denatured at high temperatures, thus the low content in this study [97]. The mashing and wort boiling temperature could have affected the β-amylase content due to the mashing temperature of 60 °C in this study [100]. In their study, De Schepper et al. [103] noted that α-amylase and β-amylase are temperature-dependent. α-amylase is inactivated at 63–71 °C and β-amylase at 54–66 °C [103]. These two enzymes are very important as α-amylase breaks complex, insoluble starch molecules into smaller, soluble molecules that are more stable thermally. α-amylase produces low molecular weight sugars, glucose, maltose, and maltotriose. β-amylase, being an unstable enzyme at high temperatures, produces only maltose. Once its activity reaches a peak, it declines and then drops at an increase in temperature [104,105,106]. The activities of these enzymes (α- and β-amylase) are relatively dependent on the temperature and time of mashing and wort boiling [103], as shown in this study. The inactivation is thus attributed to the starch hydrolyses by the two enzymes. α-amylase is an endo-acting enzyme that degrades starch during mashing and cleaving α-1,4-D-glucosidic linkages to produce oligosaccharides and limit dextrins [97,107,108]. On the other hand, β-amylase is an exo-acting enzyme, hydrolysing starch and oligosaccharide α-1,4-D-glucosidic linkages from the non-reducing end to produce maltose [109,110,111]. Thus, having enzyme-rich malt and syrup would greatly depend on the extraction temperature due to the heat-sensitive nature of the α- and β-amylases. However, boiling the BGN syrup at temperatures lower than 60 °C could increase amylase concentrations.

### 2.5. Total Polyphenols Content and Antioxidant Activities of Bambara Groundnut Speciality Malts and Syrups

Total polyphenols content and antioxidant activities of BGN speciality malts are illustrated in Table 6. There was a significant (*p* = 0.000) difference with an increase in total polyphenols and antioxidants content from 1.50 to 3.11 mg GAE/g. Moreover, the antioxidants increased, where FRAP ranged from 4.89 to 15.89 µmol AAE/g and DPPH ranged from 6.36 to 14.13 µmol TE/g. The increase in total polyphenols and antioxidants during kilning may be attributed to the extraction and release of bound phenolic compounds ((+)-catechin and ferulic acid) due to friable tissue created by kilning [10,30,112]. This friable tissue made it easy to extract the phenolic compounds better by synthesising some hydrolytic enzymes in studied grains such as barley, quinoa, millet, and sorghum [21,113,114,115,116,117,118].

The total polyphenols by Folin–Ciocâlteu reagent (FCR), antioxidant activities by ferric-reducing antioxidant power (FRAP), and 2,2-diphenyl-1-picrylhydrazyl (DPPH) assay showed an increase with the increase in kilning time and temperature. Increased antioxidant properties are contributed by the Maillard reaction products (MRPs) produced during kilning of the malting process [23]. Mainly, the roasting processes exhibit heat-induced antioxidants MRP called melanoidins [31,119]. Continuous research on antioxidants during the malting process, especially the kilning time and temperature, has clearly shown that dark speciality malt had the most significant antioxidant activities [31,120].

The BGN speciality malt syrups exhibited total polyphenols of 0.72, 0.65, 1.20, and 1.60 mg GAE/g, FRAP 2.00, 1.20, 2.42, and 4.43 µmol AAE/g, and 1.56 1.51, 2.11, and 2.96 µmol TE/g, for the base, caramel, roasted, and toasted BGN speciality malt syrups, respectively, in Table 7. There was a significant difference across the BGN speciality syrups. The total polyphenols activity in the toasted malt syrup was the highest, while the caramel malt had the lowest value.

Since Maillard reaction activities enhance the colour of the speciality malt during kilning of malt and boiling of wort, there is an increase in total polyphenols after boiling the malt extracts to produce syrup [28]. Coghe et al. [31] showed in their investigation that dark speciality malts and their extracts had the highest antioxidant activities due to higher heat application, as heat treatment is linked with an increase in antioxidant activity. The antioxidant activity of speciality malt wort increase was attributed to redox-reducing antioxidants developed during curing and roasting, giving rise to malt colour change and antiradical antioxidant activity formed during the Maillard reaction [22,121]. Samaras et al. [19] noted that the antioxidant activity of phenolic compounds and antioxidants was higher for the darkly kilned malts as Maillard reaction products increased. Maillard reaction products have antioxidant properties that influence the oxidative stability of wort [22,24,28]. However, studies have shown that malt kilned at high temperatures has the most increased antioxidant activity, contributing to higher intensities of Maillard reaction products [36,117]. Congress worts produced from vetch, green lentil, chickpea, and yellow pea malts had high phenolic and antioxidant components [74]. In the Folin–Ciocalteu, DPPH, and FRAP assays, vetch had the highest total polyphenols and antioxidants [74]. The high content of total polyphenols and antioxidants is attributed to the dark colour and hardcover characteristics of this type of legume seed, having higher flavonoids and condensed tannins, which may increase antioxidant activity [74,122]. Research works and reports have noted that legumes with dark-coloured and tough seed coats have strong antioxidant characteristics [122,123,124,125,126]. BGN is characterised by tough and coloured (black, dark brown, red, white, and speckled) varieties that could be attributed to the increased antioxidant in BGN speciality toasted malt activities in this study [124,127]. Thus, a desirable high total polyphenolic and antioxidant food product could be produced from the BGN toasted malt and syrup.

### 2.6. Total Soluble Solid of Bambara Groundnut Malt Syrups

**Figure 6:**
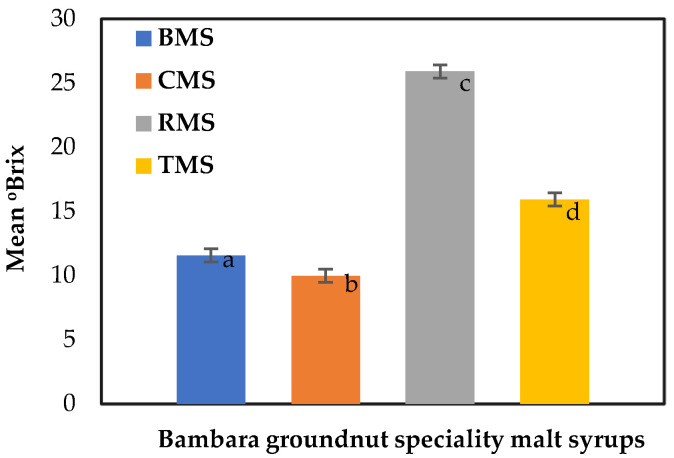
Degree Brix of Bambara speciality malt syrups. Values are the mean of triplicates ± standard deviation; mean values on the bars with different letters are significantly different (*p* < 0.05). BMS—base malt syrup, CMS—caramel malt syrup, RMS—roasted malt syrup, TMS—toasted malt syrup.

The degree Brix (°Brix) of the BGN speciality malt syrups was 11.57, 9.97, 25.90, and 15.93 °Brix, as illustrated in Figure 6. The °Brix for roasted malt syrup was the highest, indicating the highest total soluble solids content. A degree Brix (°Brix) is a gram of sucrose in 100 g of solution. The soluble solids recorded in the legume malt worts by Gasiński et al. [74], without the addition of enzyme consisting of vetch, green lentil, chickpea, and yellow pea (2.40, 1.59, 2.39, and 2.80 Plato° (≈°Brix)), were lower than the values for BGN malt syrups. Meanwhile, in the malted and unmalted rice syrups production by Ofoedu et al. [69], the °Brix was higher for the malted rice syrups, peaking at 72.10 °Brix. The high °Brix value was attributed to increased hydrolytic activity during germination and mashing by releasing more hydrolysates.

Furthermore, it was recognised that the physicochemical characteristics and quality of malts depend on the kilning duration and intensity, which will affect the mashing and wort quality [78]. The quality of the extract and malt extract syrup will add value to the production of foods by serving as a source of sweetener, flavour, colour, and enzymes [128,129]. The high total soluble content of the roasted malt syrup could be desirable in producing a non-alcoholic beverage that will add natural sweetness to the product and benefit consumers’ well-being.

### 2.7. Metabolites of the Bambara Groundnut Speciality Malts

The BGN speciality malts (base malt, caramel malt, roasted malt, and toasted malt) were profiled for metabolites, including the amino acids, sugars, sugar alcohol, organic acids, fatty acids methyl esters (FAME), and volatiles, as illustrated in the following sections.

#### 2.7.1. Amino Acid Compositions of Bambara Groundnut Speciality Malts

The amino acid of the BGN speciality malts was significantly (*p* = 0.000) different from the base, caramel, roasted to toasted except for leucine which was not significantly different across the BGN speciality malts, as shown in Table 8. The non-essential amino acids consist of aspartic acid, glutamic acid, cysteine, serine, proline, alanine, glycine, and tyrosine. Lysine was the highest amino acid for the base, caramel, and roasted malts at 61.97, 52.67, and 38.89 mg/g, respectively, while aspartic acid was the highest for toasted malt at 14.46 mg/g. On the other hand, methionine was the lowest amino acid for all BGN speciality malt types. This is because methionine, a sulphur-containing essential amino acid, is more deficient in legumes than other essential amino acids while rich in lysine [130,131,132]. However, raw BGN has a considerably high amount of methionine, ranging from 1.30 to 2.90 g/100 g compared to other legumes [133,134,135,136].

The amino acid profile for the BGN speciality malt (BM, CM, RM, and TM) showed higher amino acid contents than the raw BGN seeds. Nzelu [137] and Chinma et al. [15] noted that germination increases the amino acid content of BGN due to protease activity. However, there was a consistent decline in the amino acids of the BGN speciality malts. The decline has been attributed to different kilning temperatures and the initiation of Maillard reactions between reducing sugars and amino compounds in barley malts [138]. Samaras et al. [19] noted that the concentrations of amino acids decreased with increased heat treatment applied to barley grains in the production of speciality malts. The decrease in amino acids was also attributed to the Maillard reaction level and sugar caramelisation by Strecker degradation at higher temperatures [32,139]. This study showed that BGN speciality malts varied in amino acid concentration due to the drying conditions; hence, the base malt with the highest amino acid concentration could be optimised for production to use as functional ingredients in food and beverage production.

#### 2.7.2. Acids, Sugars, and Sugar Alcohol of Bambara Groundnut Speciality Malts

Lactic acid, a non-volatile organic acid, was present in all the BGN speciality malts, where toasted malt (0.06 mg/g) had the highest content. There was a significant (*p* = 0.000) difference in the lactic acid concentration for the base, caramel, roasted to toasted BGN speciality malts. The higher lactic acid contents have been attributed to the kilning time and temperature by [140]. Comparing two malting regimes of barley, South [141] noted that kilning time is important for final lactic acid levels in malts, where long kilning times lead to high levels of lactic acids. It was suggested that lactic acid must have been produced by dividing the grain microbes of the malt during kilning. The concentrations of the acid, sugar, and sugar alcohol in the BGN speciality malts are illustrated in Table 9.

Sugars and sugar alcohols consisting of fructose, sucrose, and myo-inositol were also present in the BGN speciality malts in appreciable concentrations, as is illustrated in Table 9. The toasted malt (0.76 mg/g) had the highest concentration of myo-inositol, while the roasted malt had a higher concentration of fructose and sucrose at 0.34 and 9.08 mg/g, respectively. However, the fructose and sucrose concentrations for the BGN speciality malts were not significantly different. The varying concentration of sugars in the BGN speciality malt was attributed to the intensity and duration of the heat applied during kilning, resulting in Maillard reaction formation and sugar caramelisation common in extremely roasted malts [19]. However, Almeida et al. [142] noted that sucrose is more abundant in the pilsner malt (base malt variety) profiled by high-performance–liquid chromatography (HPLC). However, it was suggested that the heat application during kilning increased the sugar composition of the final malt product as sugar was used as precursor for the thermally generated compounds [51]. Therefore, based on the sugar, sugar alcohol, and acid concentration of the BGN speciality malt in this study, toasted malt could be produced for its use in the production of various food products, particularly in beverage industries.

#### 2.7.3. Fatty Acids Methyl Esters (FAME) of Bambara Groundnut Speciality Malts

**Figure 7:**
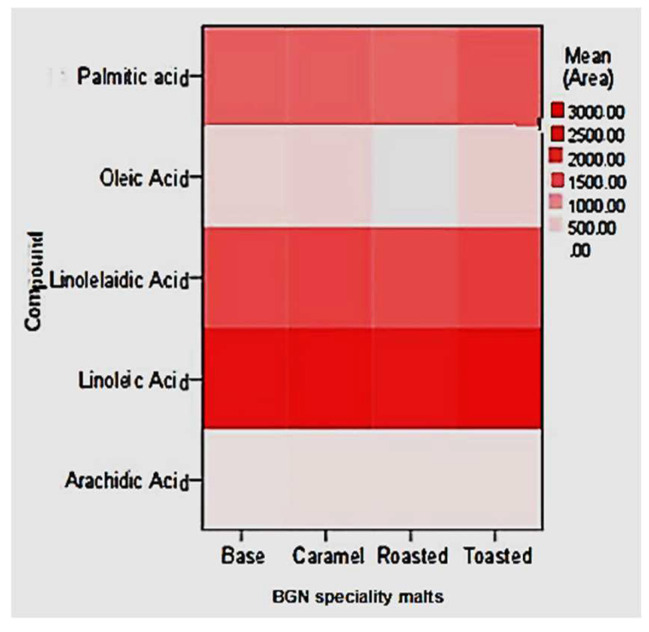
Heat plots of saturated, monosaturated, and polyunsaturated FAME of speciality Bambara groundnut malts.

The FAME identified in the base, caramel, roasted, and toasted Bambara groundnut speciality malts were palmitic, oleic, linolelaidic, linoleic, and arachidic acid, as illustrated in Figure 7. The metabolite levels on the heatmap correspond to the colour temperature, and higher temperatures indicate higher levels of FAME compounds. The BGN speciality malts exhibited FAME in different concentrations. Linoleic acid was abundant in all the BGN speciality malt types, while oleic acid was the lowest and was absent in the roasted malt.

The major fatty acid components in raw BGN are caprylic, capric, lauric, palmitic, palmitoleic, oleic, and linoleic acids [143,144]. Whereby linoleic acid was found to be the highest fatty acid in raw BGN seeds [145], which could contribute to its concentration in the speciality malts. Similar to this study, Özcan et al. [146] reported that linoleic acid content in barley malt increased during the malting process (steeping, sprouting, and drying), whereas oleic and palmitic acid content decreased. Bravi et al. [147] also noted that the linoleic acid increased in barley malt after kilning, which could be why the BGN speciality malt in this study exhibited high concentration. Furthermore, an increase in heat application to linoleic acid has been found to increase its concentration, which was attributed to varying lipids biosynthesis during the malting process [148,149,150]. Linoleic acid, oleic acid, and palmitoleic acids are essential unsaturated fatty acids necessary in human food to prevent certain heart diseases [143,151,152,153,154]. Therefore, being abundant in the BGN speciality malt across all products could benefit human health and encourage its production in large quantities.

#### 2.7.4. Volatile metabolites in Bambara groundnut speciality malts

**Figure 8:**
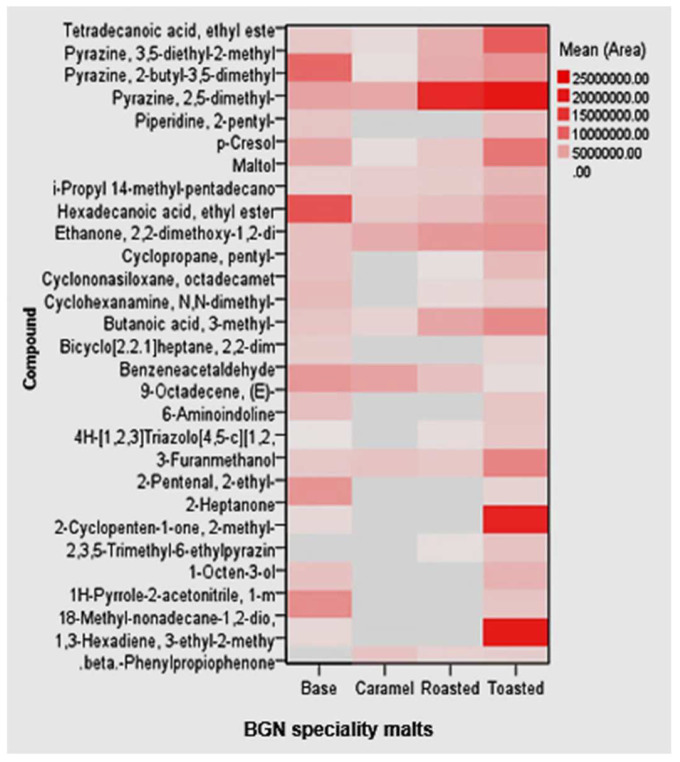
Heat plots of the volatile metabolites of Bambara groundnut speciality malts.

A total of 29 volatile metabolites were identified in the BGN speciality malts based on retention times and mass spectrometric data from MS libraries by HS-GC-FID. The volatile compounds consisted of pyrazine, furans, aldehydes, ketones, esters, and alcohols. The most abundant volatile compound in the BGN speciality malts was the pyrazine, 2,5-dimethyl, higher in the toasted malt. Conversely, the lowest volatile compound was the 2,3,5-Trimethyl-6-ethylpyrazine in all the BGN speciality malts. The volatile compounds in the speciality malts are on the heatmap illustrated in Figure 8.

The most abundant volatiles in the BGN speciality malts were the pyrazines. Pyrazines are volatile compounds with monocyclic aromatic rings with two nitrogen atoms. Foods can contain different groups of pyrazines, which consist of alkyl, methoxy, and sulphur-containing chains [155]. However, pyrazine, 2,5-dimethyl, is the most abundant in the BGN speciality malts. It is characterised by chocolate and roasted nut flavours [156]. Thus, it is an essential flavour compound in roasted food products, especially roasted coffee [157]. In addition, it is used as a flavour additive and odorant in foods such as cereals; it also occurs naturally in asparagus, green tea, crispbread, malt, raw shrimp, soya, and wheat bread [158,159,160,161]. Its high concentration in the BGN toasted malt could be attributed to the subjection to higher temperatures after initial drying of 50 °C. Methylpyrazine volatile compounds, heterocyclic volatiles, are formed by the Maillard reaction and are also common in the pyrolysis process at higher temperatures and with very low moisture contents [139,162]. The pyrolysis process thus suggested that the maltol present in the BGN speciality malts was formed in addition to Maillard reactions, which accounts for its higher concentration in toasted malt due to its low moisture and high-temperature drying.

Maltol (3-Hydroxy-2-methyl-4-pyrone), a naturally occurring organic compound used as a flavour enhancer, is found only in highly roasted speciality malts such as roasted and toasted malts [163]. Maltol is formed due to the Maillard reaction and is characterised by a sweet baked aroma typical in highly heated malts [139]. The impact of different times and temperatures applied during the caramelisation process of roasted and toasted malt developed the caramel-like flavour maltol [33]. Maltol is a safe, reliable, natural antioxidant, food preservative, and flavour [164]. It is found in baked products, red ginseng root, coffee, chicory, soybeans, bread crusts, and caramelised foods [35,165]. It has also been used in catalysis, cosmetics, pharmaceutical formulation, and food chemistry [166,167]. In addition, it can be used to treat anaemia, tumour, nerve cell oxidative stress, and kidney damage [164,168]. Studies have also shown that maltol reduced acute alcohol-induced liver injury, prevented oxidative injury through activating some signalling pathways, and prevented cisplatin-induced acute kidney injury [169,170].

The lowest volatile compound was the 2,3,5-Trimethyl-6-ethylpyrazine, mainly in roasted malt. It is a nitrogen-containing compound in the pyrazines group of volatile heterocyclic [160]. It is characterised by an earthy, nutty, roasted flavour formed during roasting at high temperatures between 135 and 250 °C [155]. It is also a chocolate enhancer used in foods containing coffee, cocoa, meat, and potatoes as a roasted flavour [171].

The volatiles in the BGN speciality malts have flavour characteristics used in the food industries to enhance and improve acceptability of food products for consumers [155,171]. These days, organic and natural labels have been gaining popularity as consumers become more aware of the ingredients in their food. Due to the high demand of consumers to eat organically grown food, the need for volatile flavours has increased, and there is a need to extract these volatiles from natural products for use in food production [171,172,173]. Thus, toasted malt with more abundant volatiles, such as maltol and pyrazine, 2,5-dimethyl, could be used for food and beverage production. Moreover, the physicochemical and biochemical characteristics of the speciality BGN malts and their syrups produced from optimum amylase malt showed good characteristics that can be incorporated into food production as ingredients or condiments.

## 3. Materials and Methods

### 3.1. Materials, Reagents, and Equipment Sources

The amylase-rich BGN malt, speciality malt, and syrups were obtained from the Department of Food Science and Technology, Cape Peninsula University, South Africa. Chemicals and reagents were of analytical standards. Alpha and beta-amylase kits were from Megazyme Ltd., Ireland. The equipment was from the Department of Food Science and Technology and Oxidative Stress Research Centre, Cape Peninsula University of Technology, Cape Town, South Africa. Equipment included the Dumas nitrogen analyser LECO CN 628 (Leco Corp., St Joseph, MI, USA). The Avanti® J-E centrifuge JSE111330 (Beckman Coulter Inc., Indianapolis, IN, USA), and Thermo Scientific MultiSkan plate reader spectrophotometer (Thermo Scientific, Waltham, MA, USA). The other equipment included the pH meter (Hanna Checker pH meter, Model HI1270), water bath, Colour Flex EZ (Model TC-P III-A, Tokyo Denshoku Co., Ltd., Tokyo, Japan), and an Excalibur Food Dehydrator (Excalibur, Sacramento, CA, USA).

### 3.2. Bambara Groundnut Speciality Malts and Syrups Physicochemical Analysis

#### 3.2.1. Colour Determination of Speciality Bambara Groundnut Malts and Syrups

Following the method of [174], the colour measurement of the Bambara groundnut speciality malts and their respective extract syrups were analysed using Colour Flex EZ (Hunter Lab, Reston, VA, USA) with daylight illumination set at D65, 10° standard observer angle, and 25 mm aperture. The standard black (L* = 8.47, a* = −0.96, b* = 2.79) and white (L* = 8.47, a* = −0.96 b* = 2.75) tiles were used for the instrument’s calibration. Five grams (5 g) of the samples in triplicate was measured into a glass sample cup (Hunter Lab 04720900, 6.4 cm) with an internal diameter of 6.4 cm following the method by [175]. The CIEL*a*b* (Commission Internationale de l’Eclairage’s) was used to measure the colour parameters, where L* is 0 = black and 100 = white, a* is −a*  =  greenness, and +a*  =  redness and b* is −b*  =  blueness and +b*  =  yellowness, respectively. As shown in Equations 1 and 2, the chroma and hue angle (h°) were calculated following the method of [176].

(1) $$C=\sqrt{a^{*2}{+b}^{*2}}$$

where C = chroma; a*^2^ = redness; b*^2^ = greenness.

(2) $$h^{o}=\tan^{-1}\left(\frac{b^{*}}{a^{*}}\right)$$

where h° = hue angle; a*^2^ = redness; b*^2^ = greenness.

#### 3.2.2. Determination of Speciality Bambara Groundnut Malts and Syrups pH

Ten milligrams (10 mg) of milled BGN speciality malts (BM, CM, RM, and TM) was separately mixed with 40 mL of distilled water in a 50 mL centrifuge tube for 5 min using a vortex mixer by following the [177] method with some differences. After mixing, the centrifuge tubes containing the mixtures were kept for 1 h at ambient temperature and centrifuged for 10 min at 1500× *g*. The supernatant’s (at room temperature) pH was measured in triplicate using a laboratory pH meter calibrated with buffers 4 and 7 (Hanna Checker pH meter, Model HI1270).

#### 3.2.3. Protein Content Determination of Bambara Groundnut Speciality Malts and Syrups

Bambara groundnut speciality malts and syrups’ crude protein was determined using the LECO CN 628 Dumas nitrogen analyser (Leco Corp, St Joseph, MI, USA). The samples in triplicate were analysed after five blanks, EDTA standard, and ProNutro control sample. The samples to the value of 0.09 mg were wrapped and tightly folded in tin foil cups, P/N: 502-186-200, and combustion was carried out in pure oxygen at a temperature of 950 °C in the reactor consisting of the combustion catalyst. A mixture of gases containing CO_2_, H_2_O, NO, and NO_2_ (carbon dioxide, water, and nitrogen) was created during the fast combustion reaction. Designated columns absorbed the gases, where oxygen was removed, and nitrogen oxides were converted into nitrogen. The remaining carbon dioxide (CO_2_) and water (H_2_O) were removed via a thermal conductivity column carried by helium gas. The nitrogen content was then measured by the Dumas Nitrogen analyser. Following the [178] method, the crude protein was calculated by multiplying the protein factor of 6.25 expressed in percentage with the measured nitrogen.

#### 3.2.4. Determination of Apparent Degree BRIX (°Brix) of Bambara Groundnut Syrups

The method of Ofoedu et al. [69] was used to measure the total soluble sugar of the syrups at a temperature of 20 °C with a handheld KERN-SOHN refractometer (KERN ORA 10 BA/BB Kern & Sohn, GmbH, Germany). First, the standardisation of the handheld refractometer was carried out with distilled water at 20 °C until the Brix value read zero. Then, one drop of each BGN syrup was dropped on the lens (sensitive surface) using plastic filling pipettes to take measurements. Finally, the total sugar contents (°Brix) were read from the refractometer scale in triplicate.

#### 3.2.5. α- and β-Amylases Activities of Bambara Groundnut Speciality Malt Determination

Following the method of [179], the alpha and beta-amylase (α- and β-amylase) activities of the BGN speciality malts and syrups were determined in triplicates. Section 1 and Section 2 detailed the determination of the α- and β-amylase enzymes through the enzymatic Ceralpha Method (K-CERA, Megazyme) and the enzymatic kit Beta-amylase (Megazyme, K-BETA3).

##### Alpha-Amylase Assay Procedure (Ceralpha Method)

The milled BGN speciality malts and syrups of 3.0 g were measured separately into 50 mL conical flasks. Twenty millilitres (20 mL) of extraction buffer solution of pH 5.4 was added to each flask and was stirred vigorously using the vortex mixer. The samples were then extracted for 20 min at 40 °C in the incubator and occasionally stirred with a vortex mixer. After extraction, 25 mL of each sample was measured into 50 mL centrifuge tubes and centrifuged with Centrifuge 5810R at 1000× *g* for 10 min. Finally, the sample extracts were separated into 25 mL centrifuge tubes for the assay procedure

The assay was carried out by measuring 0.2 mL aliquots of Megazyme unbuffered amylase HR reagent containing blocked p-nitrophenyl maltoheptaoside (BPNPG7, 54.5 mg) and thermostable α-glucosidase (125 U at pH 6.0) into 25 mL centrifuge test tubes. Then, the 0.2 mL amylase HR reagent and the sample extracts were preincubated at 40 °C for 5 min. Next, the preincubated 0.2 mL of the samples was added directly to the tubes’ bottom containing the 0.2 mL of the amylase HR reagent solution and incubated at 40 °C for 20 min. Exactly 3 mL of stopping reagent (10 g of tri-sodium phosphate in 1 L of distilled water pH adjusted to 11.0) was added immediately after incubation; the tubes were vigorously stirred using a vortex mixer. The Thermo Electron Corporation MultiSkan Spectrum was set at 400 nm against distilled water to read the absorbance of the solution in triplicate.

##### Beta-Amylase Assay Procedure (Betamyl-3 Method)

The BGN speciality malt and syrups (0.5 g) were weighed into 25 mL centrifuge tubes, and 5 mL extraction buffer containing Tris/HCl 25 mL, 1 M, pH 8.0 plus disodium EDTA of 20 mM and sodium azide of 0.02% *w*/*v* diluted in distilled water was added. The enzymes were allowed to extract for one hour at room temperature, with repeated stirring on the vortex mixer. Then, the mixtures were centrifuged using the Eppendorf Centrifuge 5810 R at 2000× *g* for 10 min. Immediately after centrifugation, 0.2 mL of the filtrate was added to 4 mL of the dilution buffer containing MES dilution buffer 48 mL, 1 M, pH 6.2 plus disodium EDTA 20 mM, BSA 10 mg/mL, and sodium azide of 0.09% w/v. The mixed solution was then used for the assay of β-amylase activities.

The assay of the β-amylase was performed by placing an aliquot of 0.2 mL of the diluted samples into the bottom of 25 mL centrifuge tubes. The tubes were preincubated at 40 °C for 5 min, and after incubation, 0.2 mL of preincubated Megazyme Betamyl-3 substrate solution (p-nitrophenyl-β-D-maltotrioside (PNPβ-G3) plus β-glucosidase (50 U)) was added. Then, stabilisers were added to each diluted sample, and the vortex mixer was used to stir the mixture. These mixtures were incubated at 40 °C for 10 min, after which 3.0 mL of the stopping reagent (10 g of Tris buffer (Megazyme cat. No. B-TRIS500)) in 900 mL of distilled water, pH adjusted to 8.5 was added, and the contents stirred with the use of the vortex mixer. The absorbance of the solution and reagent blank reading was read at 400 nm against distilled water with a Thermo Scientific MultiSkan microplate spectrophotometer.

#### 3.2.6. Total Polyphenols and Antioxidants Activities of Bambara Groundnut Speciality Malt Determination

Followed the methods of [116,180,181], the total polyphenols and antioxidants activities of the BGN speciality malts determinations were carried out with the Folin–Ciocâlteu reagent (FCR), ferric-reducing antioxidant power (FRAP), and 2,2-diphenyl-1-picrylhydrazyl (DPPH) assays, as explicated in the following three sections.

##### Total Polyphenols Content by Folin–Ciocâlteu Reagent (FCR) Assay

Five hundred milligrams (500 mg) of BGN speciality malt and syrups was measured into screw-cap tubes to determine the total polyphenols with gallic acid as the standard. The BGN speciality malt and syrups were extracted with 10 mL of 70% methanol mixed with 0.1% HCL. The samples were then centrifuged after mixing with a vortex mixer using the Eppendorf Centrifuge 5810 R at 4000× *g*, 21 °C for 5 min. The Folin–Ciocalteu assay was carried out by measuring 25 mL of the decanted liquids and mixing it with 125 µL of 0.2 M Folin–Ciocalteu reagent and 100 µL of 7.5% Na_2_CO_3_ solution in 96-well transparent plate. The absorbance was read in triplicate with a Thermo Scientific MultiSkan microplate spectrophotometer reader (734 nm at 25 °C) after a 2 h incubation period. The standard calibration curve was constructed with 40 mg gallic acid (Sigma Cat Nr: G7384). The results were expressed as mg Gallic acid equivalents (GAE)/g).

##### Antioxidant Activities by Ferric-Reducing Antioxidant Power (FRAP) Assay

Bambara groundnut speciality malt and syrups of 500 mg were weighed into 50 mL screw-cap tubes. Ten millilitres of 70% methanol (containing 0.1% HCl) was added to the samples in the screw-cap tubes. The samples were mixed with a vortex, then centrifuged at 4000 rpm for 5 min and the supernatants (10 μL each) were pipetted into microplate wells in triplicates. Three hundred microlitres (300 µL) of the FRAP reagent was added to each sample in the microplate wells. Ascorbic acid was the standard, and distilled water was the blank. The samples were incubated for 30 min at 37 °C, and absorbance was read at 593 nm. The Thermo Scientific MultiSkan microplate spectrophotometer was used for reading absorbance. The results were expressed as mg ascorbic acid equivalents (AAE)/g.

##### Antioxidant Activities by 2,2-Diphenyl-1-picrylhydrazyl (DPPH) Assay

The Bambara groundnut speciality malts and their syrup free radical scavenging ability were determined using the DPPH radical (25 mg/L) in 70% methanol. Each of the samples was mixed with 0.275 mL DPPH solutions. The samples and standards were incubated at 37 °C for 30 min in the dark, and absorbance reactions were read at 517 nm. The Thermo Scientific MultiSkan microplate spectrophotometer was used for reading absorbance. The standard was Trolox, and results were expressed as µmole Trolox/g.

#### 3.2.7. Metabolite Profiling of Bambara Groundnut Speciality Malt

The metabolite profiling was carried out on the Bambara groundnut speciality malts. The sugars, sugar alcohols, organic acids, and amino acids were profiled by capillary gas chromatography–mass spectrometry (GCMS) [182,183,184,185,186]. A gas chromatography–flame ionisation detector (GC-FID) was used to analyse the fatty acid methyl esters (FAME) [187]. A headspace gas chromatography–flame ionisation detector (GC-FID) was used to analyse the fatty acid methyl esters (FAME) and volatile compounds [188].

##### Determination of Fatty Acids Methyl Esters (FAME) and Hydrocarbons by Gas Chromatography–Flame Ionisation Detection (GC-FID)

The analysis of fatty acid and hydrocarbons was carried out by extracting and converting the BGN speciality malt lipids into fatty acid methyl esters (FAME). The extraction was carried out using diethyl ether and petroleum ether in methanol. A model Agilent 7890A gas chromatography (GC) coupled with Flame Ionisation Detection (GC-FID) was used for detection according to the [188] method, 996.06 with some modifications.

The BGN speciality malts of 1.5 mg were weighed into the separate 70 mL test tubes to digest. The tube’s contents were thoroughly mixed with 100 mg of pyrogallic acid, 2 mL internal standard solution of 5 mg/mL undecanoic acid dissolved in hexane, and 2 mL ethanol. Immediately after mixing, 10 mL of 32% HCL was mixed into each tube. The tubes were then placed in a 70–80 °C water bath for 40 min, and the contents were mixed every 10 min. After digestion, the tubes were removed and allowed to cool to room temperature. The 25 mL diethyl ether was added to each tube and shaken for 5 min for extraction. Petroleum ether of 25 mL was further added and shaken for 5 min. After separating the two layers, the clear upper layer was decanted into 150 mL beakers, and ether was evaporated in the fume hood to dryness.

Derivatisation of the samples was carried out by reconstituting the residues in 3 mL chloroform and diethyl ether. The solutions were transferred into 10 mL tubes and evaporated under the nitrogen stream to dry. Immediately after drying, 2 mL of 2% H_2_SO_4_ in methanol reagent and 1 mL toluene were added. The tubes were tightly closed and placed in the incubator at 100 °C for 45 min, then cooled to room temperature. After cooling, 5 mL distilled water and 1 mL hexane were added and thoroughly shaken, using the vortex mixer for 1 min. The layers were left to separate, and the top layers were carefully transferred to 20 mL test tubes. Approximately 1 g anhydrous Na_2_SO_4_ was added to each tube to have a clear solution. The clear solutions were then transferred into 2 mL clear vials, and GC analysis was carried out. Fatty acids were identified by comparing their retention times to the retention times of the standard.

##### Sugars, Acids, and Sugar Alcohols Determination by Gas Chromatography–Mass Spectrometry (GC-MS)

Sugars, sugar alcohols, and organic acids were analysed using GCMS by measuring 1 mL of 70% methanol (MeOH), then adding approximately 100 mg of the BGN speciality malts and extracting at 45 °C in the oven for 3 hours. The extracted samples of 130 µL were dried completely with a gentle stream of nitrogen and derivatised with 100 µL of methoxamine at 40 °C for 2 h. Then, 30 µL of N, O-Bis (trimethylsilyl) trifluoroacetamide (BSTFA) was added and derivatised at 60 °C for 30 min. Finally, the samples were transferred into 2 mL GC vials, and 1 µL was injected onto the GC-MS in spit-less mode.

Separation was performed on a gas chromatograph (Trace 1300, Thermo Fisher Scientific S.p.A., Strada Rivoltana 20090 Rodano-Milan, Italy) coupled with a mass spectrometer (TSQ 8000, Thermo Scientific). The carbohydrates were separated on a non-polar capillary column Rxi-5Sil MS (30 m, 0.25 mm ID, 0.25 µm film thickness). Helium was used as the carrier gas at a 1 mL/min flow rate. The injector temperature was maintained at 250 °C. The oven temperature was 80 °C for 1 min and was ramped up to 300 °C at a rate of 7 °C/min and held for 2 min.

##### Amino Acids Determination by Gas Chromatography–Mass Spectrometry (GC-MS)

Following the method of [189] with a little difference, 3 mL of 6M HCl was added to ca. 500 mg of the BGN speciality malts (BM, CM, RM, and TM). They were hydrolysed for 24 h at 110 °C, cooled down to room temperature, and diluted at a ratio of 1:9 with 70% methanol (*v*/*v*). Next, 100 µL was transferred into a 2 mL tube and dried completely under a gentle stream of nitrogen. Then, the samples were reconstituted and derivatised with 30 µL silylation reagent N-tert-butyldimethylsilyl- N-methyl trifluoroacetamide (MTBSTFA) and 100 µL acetonitrile at 100 °C for 1 h. After which, they were cooled down to room temperature and injected into the GC-MS instrument for analysis.

Component separation was performed on a gas chromatograph (Trace1300, Thermo Fisher Scientific S.p.A., Strada Rivoltana 20090 Rodano-Milan, Italy) coupled to a TSQ8000 mass spectrometer (Thermo Scientific). The GC-MS system was connected to a TriPLUS autosampler. Amino acids were separated on a capillary column Rxi-5Sil MS (30 m, 0.25 mm ID, 0.25 µm film thickness). Helium was used as the carrier gas at a 1 mL/min flow rate, and the injector temperature was maintained at 250 °C. In addition, 1 µL of the sample was injected in spit-less mode. The oven temperature was programmed to 100 °C for 1 min and ramped up to 300 °C at a rate of 15 °C/min and held for 6 min. The Agilent mass spectrometer detector (MSD) was operated in scan mode, and the source and quad temperatures were maintained at 250 °C and 150 °C, respectively. The transfer line temperature was maintained at 250 °C. The mass spectrometer was operated under electron impact (EI) mode at ionisation energy of 70 eV by scanning from 35 to 650 *m*/*z*.

##### Volatile Compounds Determination by Headspace Gas Chromatography–Mass Spectrometry (HS-GC-MS)

The headspace gas chromatography–mass spectrometry (HS-GC-MS) analyses were performed using a model Agilent 7890B Gas Chromatography–5977A coupled with a Mass Spectrometer detector system (Agilent Technologies, Santa Clara, CA, USA) with a split-less injector that is suitable for GC analysis by following the method of [187] with some differences. The Agilent J&W GC HP-5ms capillary column of 30 m × 0.25 mm × 0.25 µm was used to separate the volatiles. The carrier gas was helium, with a 0.6 mL/min flow rate. Two hundred and fifty microlitres of the speciality malts volume was injected with a split ratio of 50:1 and weighed into 10 mL glass headspace vials covered with silicon septum with a purge flow of 3 mL/min and screw-capped. The oven temperature was 50 °C, held for 5 min, increased at 10 °C/min to 200 °C and held for 5 min with a running time of 25 min. The injector temperature, pressures, and volume were set at 240 °C, 2.6149 psi, and 250 μL, respectively. The incubation temperature and time were set at 120 °C and 300 s, respectively. The samples were then run concurrently.

The compounds were identified through Wiley mass spectral (MS) library and Golm metabolome database search. The volatile compounds identification was performed by comparing the mass spectra with the spectra of the reference compounds in both the Wiley MS library and was verified based on mass spectra obtained from the literature. The volatile results were provided based on the compounds’ quality and peak area counts.

### 3.3. Identification of Metabolite Compounds

Identification of BGN speciality malt constituents was performed by comparing the retention times and mass spectra with reference compounds. Moreover, it was conducted by comparing mass spectra with the entries of the National Institute of Standards and Technology mass spectra library NIST02 and the GOLM metabolome database [183,184,186,190].

### 3.4. Statistical Data Analysis

All results were reported as mean ± standard deviation of three independent trials. Multivariate analysis of variance (MANOVA) was used to establish differences between treatments. Duncan’s multiple range test was used to separate means where significant differences existed ((SPSS version 26.0, IBM Corporation, Armonk, NY, USA)). Kruskal–Wallis test was used to test the distribution of protein when normality is violated to determine the mean differences between treatments.

## 4. Conclusions

This study successfully produced speciality Bambara groundnut malts and their corresponding syrups from the amylase-rich green BGN malts steeped at 36 h and sprouted at 96 h. The speciality malts and syrups exhibited colours desirable in the food industries, which could be used to impact different shades of colours as ingredients in product formulation in baked goods. The BGN speciality malt syrups exhibited a similar pH range to malted barley syrup, making it a functional ingredient in the beverage industries. Bambara groundnut speciality roasted malt and toasted malt syrup exhibited favourable protein concentration compared to base and caramel malts, which could benefit human health when consumed. The enzyme activities were affected by heat application during malt kilning and extract boiling due to the heat-sensitive nature of the α- and β-amylases. However, boiling at temperatures lower than 60 °C could be recommended for the production of BGN syrups with higher amylase concentrations. The toasted malt and its syrup exhibited the highest total polyphenolic and antioxidant activities, which could make it a desirable functional food product ingredient. The °Brix of the roasted malt syrup was the highest, which could be a desirable attribute in producing non-alcoholic beverages by adding natural sweetness to the product, and which can be of benefit to consumers who are health-conscious. The profile of metabolite components in the speciality BGN malt included amino acids, fatty acid methyl esters, sugars, sugar alcohol, acid, and volatiles. The metabolites identified in the BGN speciality malt could add value to the sensory properties and nutritional and functional characteristics of BGN seeds. Thus, the speciality Bambara groundnut malt possesses components that can be incorporated into human diets for their health benefits. Hence, its use in the food and beverage industries should be encouraged.

## Figures and Tables

**Table 1:** Colour characteristics of Bambara groundnut speciality malt ^1^.

BGN Speciality Malt	L*	a*	b*	C	h°
Base	74.12 ± 0.29 ^a^	3.96 ± 0.71 ^a^	11.85 ± 1.24 ^a^	12.50 ± 1.30 ^a^	71.54 ± 2.63 ^a^
Caramel	74.24 ± 0.26 ^a^	4.76 ± 0.86 ^a^	15.31 ± 0.26 ^a^	16.04 ± 0.33 ^a^	72.74 ± 2.98 ^a^
Roasted	63.91 ± 0.45 ^b^	9.87 ± 0.52 ^b^	22.41 ± 2.55 ^b^	24.51 ± 2.23 ^b^	66.05 ± 3.06 ^b^
Toasted	45.98 ± 0.27 ^c^	16.44 ± 0.63 ^c^	22.68 ± 2.99 ^b^	28.03 ± 2.80 ^b^	53.90 ± 2.48 ^c^

^1^ Values are the mean of triplicates ± standard deviation; mean values in the same column with different superscript letters are significantly different (*p* ≤ 0.05). BGN—Bambara groundnut, L*—lightness; a*—redness, b*—yellowness, C—chroma, h°—hue angle.

**Table 2:** CIE L*a*b*, chroma, and hue for the Bambara groundnut speciality malt syrups ^1^.

Colour Characteristics	Bambara Groundnut Speciality Malt Syrups			
	Base Malt	Caramel Malt	Roasted Malt	Toasted Malt
L*	49.56 ± 0.15 ^a^	52.77 ± 0.07 ^b^	41.43 ± 0.32 ^c^	28.55 ± 0.69 ^d^
a*	8.07 ±1.63 ^a^	7.78 ± 1.46 ^a^	9.51 ± 2.64 ^a^	2.52 ± 0.99 ^b^
b*	15.82 ± 1.41 ^a^	20.28 ± 0.76 ^b^	16.22 ± 3.12 ^a^	8.30 ± 1.40 ^c^
Chroma	17.82 ± 1.10 ^a^	21.75 ± 0.70 ^b^	19.04 ± 1.75 ^a^	8.68 ± 1.62 ^c^
Hue angle	62.94± 5.10 ^ab^	69.03 ± 3.90 ^ab^	59.20 ± 11.47 ^a^	73.53 ± 3.66 ^b^

^1^ Values are mean ± standard deviation of triplicate values; mean values in the same column with different superscript letters are significantly different (*p* ≤ 0.05). L*—lightness; a*—redness, b*—yellowness.

**Table 3:** pH characteristics of Bambara groundnut speciality malt syrups ^1^.

Bambara Groundnut Speciality Malt Syrup	pH
Base malt	5.52 ± 0.06 ^a^
Caramel malt	5.13 ± 0.04 ^b^
Roasted malt	5.46 ± 0.03 ^a^
Toasted malt	5.71 ± 0.01 ^c^

^1^ Mean values on the column with different superscripts are significantly different (*p* ≤ 0.05).

**Table 4:** Bambara groundnut specialty malts α and β-amylase activities ^1^.

BGN Speciality Malt	Alpha-Amylase	Beta-Amylase
Base	1.01 ± 0.01 ^a^	0.11 ± 0.00 ^a^
Caramel	0.21 ± 0.00 ^b^	0.10 ± 0.00 ^b^
Roasted	0.29 ± 0.00 ^c^	0.10 ± 0.00 ^c^
Toasted	0.15 ± 0.00 ^d^	0.06 ± 0.00 ^d^

^1^ Values are the mean of triplicates ± standard deviation; values in the same column with different superscripts are significantly different (*p* ≤ 0.05).

**Table 5:** Amylase activities of Bambara groundnut speciality malt syrups ^1^.

BGN Speciality Malt Syrup	α-Amylase	β-Amylase
Base malt	0.39 ± 0.00 ^a^	0.14 ± 0.00 ^a^
Caramel malt	0.31 ± 0.00 ^b^	0.13 ± 0.00 ^b^
Roasted malt	0.30 ± 0.00 ^b^	0.15 ± 0.00 ^c^
Toasted malt	0.31 ± 0.00 ^c^	0.21 ± 0.00 ^d^

^1^ Values are the mean of triplicates ± standard deviation; values in the same column with different superscripts are significantly different (*p* ≤ 0.05).

**Table 6:** Total polyphenols and antioxidant activities of Bambara groundnut speciality malts ^1^.

Bambara Groundnut Speciality Malt	Polyphenol (mg GAE/g)	FRAP (µmol AAE/g)	DPPH (µmol TE/g)
Base	1.50 ± 0.09 ^a^	4.89 ± 0.30 ^a^	6.36 ± 0.05 ^a^
Caramel	1.55 ± 0.07 ^a^	5.86 ± 0.23 ^a^	6.81 ± 0.92 ^a^
Roasted	3.11 ± 0.25 ^b^	15.39 ± 0.56 ^b^	14.13 ± 0.13 ^b^
Toasted	2.86 ± 0.23 ^b^	15.89 ± 0.90 ^b^	13.70 ± 1.22 ^b^

^1^ Values are the mean of triplicates standard deviation; values in the same column with different superscripts are significantly different (*p* ≤ 0.05). GAE—gallic acid equivalent, AAE—ascorbic acid equivalents, TE—Trolox equivalent.

**Table 7:** Total polyphenols and antioxidant activities of Bambara speciality malt syrups ^1^.

BGN Speciality Malt Syrup	Total Polyphenols (mg GAE/g)	FRAP (µmol AAE/g)	DPPH (µmol TE/g)
Base malt	0.72 ±0.04 ^a^	2.00 ± 0.14 ^b^	1.56 ± 0.13 ^a^
Caramel malt	0.65 ± 0.03 ^a^	1.20 ± 0.02 ^a^	1.51 ± 0.13 ^ab^
Roasted malt	1.20 ± 0.05 ^b^	2.42 ± 0.05 ^c^	2.11 ± 0.30 ^b^
Toasted malt	1.60 ± 0.19 ^c^	4.43 ± 0.18 ^d^	2.96 ± 0.49 ^c^

^1^ Values are the mean of triplicates ± standard deviation; values in the same column with different superscripts are significantly different (*p* ≤ 0.05). GAE—gallic acid equivalent, AAE—ascorbic acid equivalents, TE—Trolox equivalent.

**Table 8:** Amino acids concentrations of Bambara groundnut speciality malts ^1^.

Essential Amino Acid	Amino Acids Concentration (mg/g)			
	Base Malt	Caramel Malt	Roasted Malt	Toasted Malt
Lysine	61.97 ± 1.17 ^a^	52.67 ± 0.17 ^b^	38.89 ± 0.40 ^c^	10.72 ± 0.82 ^d^
Threonine	15.385 ± 0.05 ^a^	12.93 ± 0.08 ^b^	13.55 ± 0.11 ^c^	9.90 ± 0.01 ^d^
Phenylalanine	13.99 ± 0.15 ^a^	11.25 ± 0.01 ^b^	12.81 ± 0.18 ^c^	9.16 ± 0.51 ^d^
Valine	12.47 ± 0.31 ^a^	11.95 ± 0.06 ^b^	12.14 ± 0.01 ^ab^	7.91 ± 0.08 ^c^
Leucine	11.91 ± 0.23 ^a^	10.41 ± 0.10 ^a^	10.45 ± 0.54 ^a^	11.26 ± 1.42 ^a^
Isoleucine	10.60 ± 0.08 ^a^	9.97 ± 0.02 ^b^	8.46 ± 0.01 ^c^	7.25 ± 0.10 ^d^
Methionine	4.52 ± 0.04 ^a^	4.38 ± 0.04 ^b^	4.24 ± 0.06 ^c^	1.92 ± 0.05 ^d^
**Non-Essential Amino Acid**				
Aspartic acid	27.45 ± 0.22 ^a^	21.84 ± 0.05 ^b^	23.00 ± 0.07 ^c^	14.46 ± 0.25 ^d^
Glutamic acid	22.24 ± 0.06 ^a^	19.98 ± 0.01 ^b^	21.44 ± 0.35 ^c^	13.66 ± 0.04 ^d^
Cysteine	22.34 ± 0.01 ^a^	12.94 ± 0.25 ^b^	15.38 ± 0.12 ^c^	7.23 ± 0.36 ^d^
Serine	13.10 ± 0.06 ^a^	10.51 ± 0.00 ^b^	11.59 ± 0.16 ^c^	11.75 ± 0.06 ^c^
Proline	13.14 ± 0.10 ^a^	11.62 ± 0.13 ^b^	11.49 ± 0.52 ^b^	7.16 ± 0.14 ^c^
Alanine	7.87 ± 0.13 ^a^	7.35 ± 0.01 ^b^	7.40 ± 0.23 ^b^	4.05 ± 0.14 ^c^
Glycine	7.42 ± 0.01 ^a^	6.81 ± 0.03 ^b^	5.73 ± 0.03 ^c^	2.71 ± 1.93 ^d^
Tyrosine	4.73 ± 0.02 ^a^	4.26 ± 0.04 ^b^	4.02 ± 0.06 ^c^	3.06 ± 0.02 ^d^

^1^ Values are the mean of duplicates’ standard deviation; values in the same row with different superscripts are significantly different (*p* ≤ 0.05).

**Table 9:** Acid, sugars, and sugar alcohol concentration of speciality Bambara groundnut malts ^1^.

Acid, Sugar, and Sugar Alcohol (mg/g)	Bambara Groundnut Speciality Malts			
	Base	Caramel	Roasted	Toasted
Lactic acid	0.04 ± 0.00 ^ab^	0.01 ± 0.00 ^a^	0.03 ± 0.00 ^ab^	0.06 ± 0.00 ^b^
Fructose	0.02 ± 0.00 ^a^	0.07 ± 0.00 ^a^	0.34 ± 0.03 ^b^	0.02 ± 0.00 ^a^
Sucrose	4.77 ± 1.10 ^a^	5.27 ± 1.50 ^a^	9.08 ± 3.10 ^b^	6.33 ± 0.70 ^ab^
Myo-inositol	0.04 ± 0.00 ^a^	0.22 ± 0.10 ^ab^	0.47 ± 0.10 ^bc^	0.76 ± 0.40 ^c^

^1^ Values are the mean of duplicates ± standard deviation, values in the same row with different superscripts are significantly different (*p* ≤ 0.05).

## Data Availability

No additional data were generated other than the data reported in the manuscript.

## Sample Availability

Not available.
